# Inception of electronic damage of matter by photon-driven post-ionization mechanisms

**DOI:** 10.1063/1.5090332

**Published:** 2019-04-05

**Authors:** W. Błachucki, Y. Kayser, J. Czapla-Masztafiak, M. Guo, P. Juranić, M. Kavčič, E. Källman, G. Knopp, M. Lundberg, C. Milne, J. Rehanek, J. Sá, J. Szlachetko

**Affiliations:** 1 Institute of Physical Chemistry, Polish Academy of Sciences, 01-224 Warsaw, Poland; 2 Physikalisch-Technische Bundesanstalt, 10587 Berlin, Germany; 3 Institute of Nuclear Physics, Polish Academy of Sciences, 31-342 Kraków, Poland; 4 Department of Chemistry–Ångström Laboratory, Uppsala University, 75120 Uppsala, Sweden; 5 Paul Scherrer Institute, CH-5232 Villigen-PSI, Switzerland; 6 Jozef Stefan Institute, SI-1000 Ljubljana, Slovenia

## Abstract

“Probe-before-destroy” methodology permitted diffraction and imaging measurements of intact specimens using ultrabright but highly destructive X-ray free-electron laser (XFEL) pulses. The methodology takes advantage of XFEL pulses ultrashort duration to outrun the destructive nature of the X-rays. Atomic movement, generally on the order of >50 fs, regulates the maximum pulse duration for intact specimen measurements. In this contribution, we report the electronic structure damage of a molecule with ultrashort X-ray pulses under preservation of the atoms' positions. A detailed investigation of the X-ray induced processes revealed that X-ray absorption events in the solvent produce a significant number of solvated electrons within attosecond and femtosecond timescales that are capable of coulombic interactions with the probed molecules. The presented findings show a strong influence on the experimental spectra coming from ionization of the probed atoms' surroundings leading to electronic structure modification much faster than direct absorption of photons. This work calls for consideration of this phenomenon in cases focused on samples embedded in, e.g., solutions or in matrices, which in fact concerns most of the experimental studies.

## INTRODUCTION

Studies on the structure of matter done with 3rd and 4th generation radiation X-ray sources, especially those of complex systems, have always been accompanied by concerns about the presence of deleterious processes induced by the incident beam.^1^ Radiation damage manifests itself through different effects like electronic structure change, bond breaking, Coulomb explosion, and structural changes. A few methodologies have been proposed to mitigate the beam damage, such as sample circulation^2^ and cryocooling techniques.^3^ However, the most significant development for suppressing the radiation damage has been the shortening of the X-ray pulse duration to tens of femtoseconds, which pioneered the growth of the so called “probe-before-destroy” approach.^4,5^ The approach allows probing molecular systems before the radiation damage causes observable changes in them. This development capitalizes in the advent of extremely bright ultrafast X-ray free-electron laser (XFEL) facilities, which are able to deliver femtosecond-short X-ray pulses reaching peak intensity on a target of up to ∼10^20^ W/cm^2^, and photon fluxes of about 10^34^ photon/(s cm^2^).^6^ XFELs are used in research on fundamental processes in atomic and radiation physics^7,8^ and in ultrafast time-resolved measurements of more complex systems and molecular processes.^9^ It was demonstrated, mainly by means of X-ray diffraction, that structural information can be retrieved devoid of beam induced damage, typically considered in terms of the Coulomb explosion, for a wide range of pulse durations up to about 50 fs and a photon flux on the order 10^32^ photon/(s cm^2^).^5^ However, it has been also shown that the impact of the incident radiation on the sample happens significantly faster when one considers electronic structure-related modifications,^10,11^ which occur within the core-hole lifetime, i.e., at sub-10 fs timescales.

The electrons bound in an atom can occupy different atomic energy levels. The removal of one electron, through interaction with an X-ray photon or with a free charged particle, results in a vacancy on one of the energy levels and leaves the atom in an excited state. The atom goes back to the ground state through different atomic processes that may give rise to emission of electromagnetic radiation. The spectral distribution of X-rays emitted in these processes is the scope of the discipline called “X-ray emission spectroscopy” (XES).^12^ In “nonresonant X-ray emission spectroscopy” (NXES), a vacancy in the electronic structure is created through the removal of a bound electron by an X-ray photon having energy fairly larger than the binding energy. The incident photon beam does not need to be monochromatic. Following the excitation, the ground state is reached through fluorescence decays, electron deexcitations resulting in emission of radiation, and Auger decays, electron deexcitations leading to ejection of another electron from an atom. NXES provides an element-specific tool to determine the energy differences between atomic energy levels and the density of occupied electronic states. It grants the access to the study the fluorescing atoms' surrounding, in particular, the ligand orbitals and bond distances (see, as example previously published reports^13,14^), and, owing to the penetrating properties of X-ray radiation, its sensitivity is not limited to the target's surface but extends to the material's bulk.

This paper reports on a nonresonant X-ray emission spectroscopy (NXES) study of a [Fe(CN)_6_]^4–^ complex in aqueous solution irradiated by an XFEL beam with 30 fs-short X-ray pulses and different photon fluxes from 5.75 × 10^30^ to 244 × 10^30^ photon/(s cm^2^). A monotonic change in the Fe Kβ emission spectrum with increasing photon flux was found. This finding cannot be related to the metal-site direct absorption, which may be considered only as a higher order contribution. Detailed investigations of the X-ray induced processes revealed that X-ray absorption by water molecules generated free electrons [high energy photoelectrons and low energy Auger electrons (secondary electrons)] from the oxygen atoms within a few fs.^15,16^ Note that herein free electron term is broadly used to describe electrons that are ejected from solvent molecules upon interaction with X-ray beam. The energetic free electrons are able to ionize the complex metal-center and to start a cascade of Auger decays creating multiple electron hole states of iron atoms. This leads to a change in the iron oxidation state but leaves the complex structure unperturbed over the course of the pulse duration. This study reveals additional strong contribution to the probed atoms' electronic structure modification much faster than direct photoabsorption. This contribution originates from the ionization of the probed atoms' environment and must be taken into consideration in most of the studies, in particular where the studied material is enclosed in solution or in soft matrices.

## EXPERIMENT

**FIG. 1.**
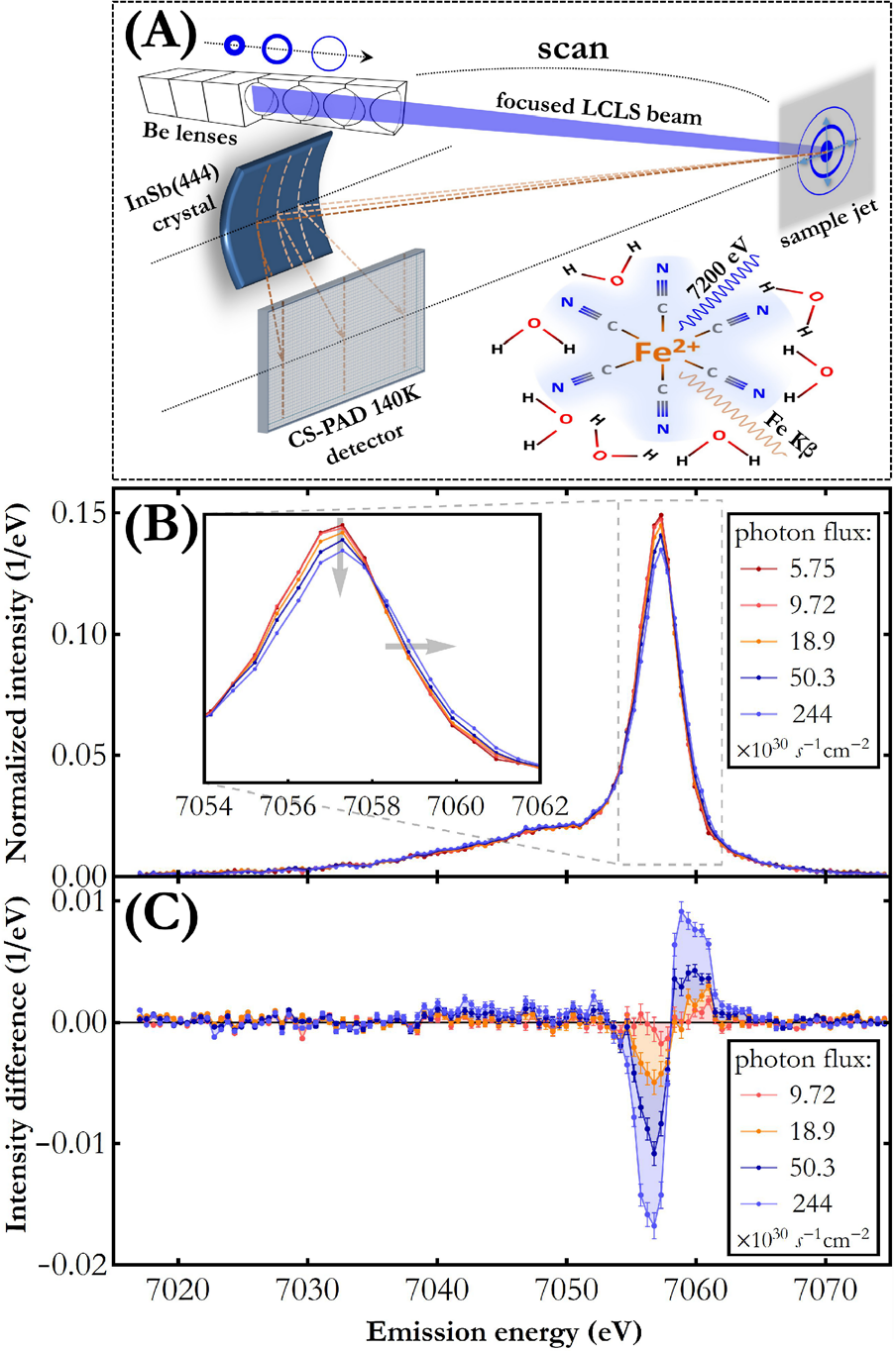
(a) Schematic of the experimental setup. Varying the distance between the movable Be lens stack and the sample allowed adjusting the incident photon flux. (b) Fe Kβ X-ray emission spectra measured for a Fe(CN)_6_/H_2_O solution for different incident photon fluxes. The spectra were normalized to unit area for integrated area difference (IAD) analysis.^23^ This normalization assumes a constant 1*s* shell ionization cross section and 3*p* → 1*s* transition fluorescence yield for different charge states. The monotonic change with rising photon flux is indicated by the arrows in the inset. The intensity measurement uncertainty does not exceed 2% in the energy range 7054–7062 eV. (c) Fe Kβ difference spectra resulting from subtraction of the consecutive spectra from the one measured for the lowest photon flux.

The experiment was carried out at the X-ray pump-probe (XPP) end-station of the Linac Coherent Light Source (Menlo Park, USA) XFEL. The outline of the experimental setup is shown in Fig. 1(a). A 0.1 M potassium hexacyanoferrate(II) trihydrate {K_4_[Fe(CN)_6_^·^3H_2_O] from Sigma-Aldrich, ≥99.95% pure} in distilled water was circulated through a liquid jet system with a 100 *μ*m-thick jet nozzle (total volume 250 ml).^17^ The flow speed of the jet was 6.5 m/s. The experiment explored nominal capabilities of the Linac Coherent Light Source (LCLS) machine and the XPP experimental station in the self-amplified spontaneous emission (SASE) mode. The jet was irradiated at the repetition rate of 120 Hz with XFEL pulses of 30 fs-duration and ∼4 × 10^11^ photons each. The photon energy was 7200 eV, which is 88 eV above the Fe 1*s* edge binding energy. The X-ray beam was focused by means of a movable Be lens stack which allowed us to vary the beam spot size on the jet in the range 6–241 *μ*m^2^ and, thus, to change the incident photon flux. For five different photon fluxes in the range 5.75–244 × 10^30^ photon/(s cm^2^), the Fe Kβ X-ray emission spectrum was measured in a shot-to-shot mode with a wavelength-dispersive X-ray spectrometer operated in the von Hamos geometry.^18^ A cylindrically bent InSb(444) crystal with 25 cm-radius of curvature and a Cornell-SLAC hybrid Pixel Array Detector (CSPAD) of 140 kilopixels^19^ were used. Details about the spectrometer, acquisition mode, and resolution can be found elsewhere.^20^ The point resolution of the von Hamos setup used is 0.5 eV. For each of the photon fluxes (five variations in total), the measured Fe Kβ XES spectra were averaged over 28 000–38 000 pulses.

## RESULTS

### Metal center oxidation state dependence on incident X-ray beam flux

The results of the Fe Kβ X-ray emission spectrum measurement for different photon fluxes are presented in Fig. 1(b). The Kβ X-ray emission signal originates from the 3*p* → 1*s* electron transitions which are very sensitive to the (3*p*,3*d*) exchange coupling and, thus, to the 3*d* population as well as to the relative orientation of the 3*d* and 3*p* electrons' spins. The spectra shown are dominated by the Fe Kβ_1,3_ main line at around 7057 eV arising from the deexcitation to the final state in which unpaired 3*p* electrons' spin is parallel to the 3*d* electrons' spin^21,22^ (the so called “high-spin” state configuration). The much weaker feature at about 7048 eV is the Fe Kβ′ line induced by the deexcitation to the final state in which unpaired 3*p* electrons' spin is antiparallel to the 3*d* electrons' spin (the so called “low-spin” state configuration). The Fe Kβ′ line is on the main line's low energy tail together with many weaker resonances caused by the valence electrons' spin flipping in response to the 3*p* → 1*s* deexcitations (the so-called “shake-up” transitions). These resonances are not resolved in the measured spectra due to their low transition probabilities and small energy shifts.

As shown in Fig. 1(b), increasing incidence photon fluxes result in an altered shape of the Fe Kβ emission spectral curve and cause a monotonic shift along the energy axis to higher values as well as a decrease in the main line's peak intensity. Figure 1(c) clearly shows that an increase in incident photon flux by a factor of 1.7 induces significant variations in the X-ray emission signal. Careful analysis of the spectra averaged over smaller sets of single shot spectra (counting, e.g., 1, 5, 10, 20 single shot spectra) revealed the same direction of changes for different photon fluxes. This implies that, under the experimental conditions achieved in this work, sample damage and probe occurred during a single X-ray pulse irradiation. We wish to stress that the liquid jet sample was refreshing shot by shot and the effect of Fe Kβ emission spectrum change vanished whenever the beam focus was moved away from the target jet (i.e., spot size on the sample increased). This confirms that the detected changes are exclusively related to incidence X-ray flux per single shot and not to any long time scale, or other, experimental effects.

The direction of observed spectral changes is similar to that reported by Zhang *et al.*^24^ for the Kβ emission from Fe atoms in [Fe(2,2′-bipyridine)_3_]^2+^. The work mentioned, however, was an optical pump-X-ray probe time-resolved study where the temporal modulation of Fe Kβ X-ray emission spectrum originated from the optically induced metal-to-ligand charge transfer leading to the molecule's spin change. In the present work, only an XFEL pulse irradiates the target and the dependence of the Fe Kβ emission signal on incident photon flux is observed. The incident beam flux is much too small to induce nonlinear effects and changes in the Fe Kβ emission are already observed at a photon flux three orders of magnitude below the sequential photoionization regime.^25^

### X-ray pulse interaction with the solvent

**FIG. 2.**
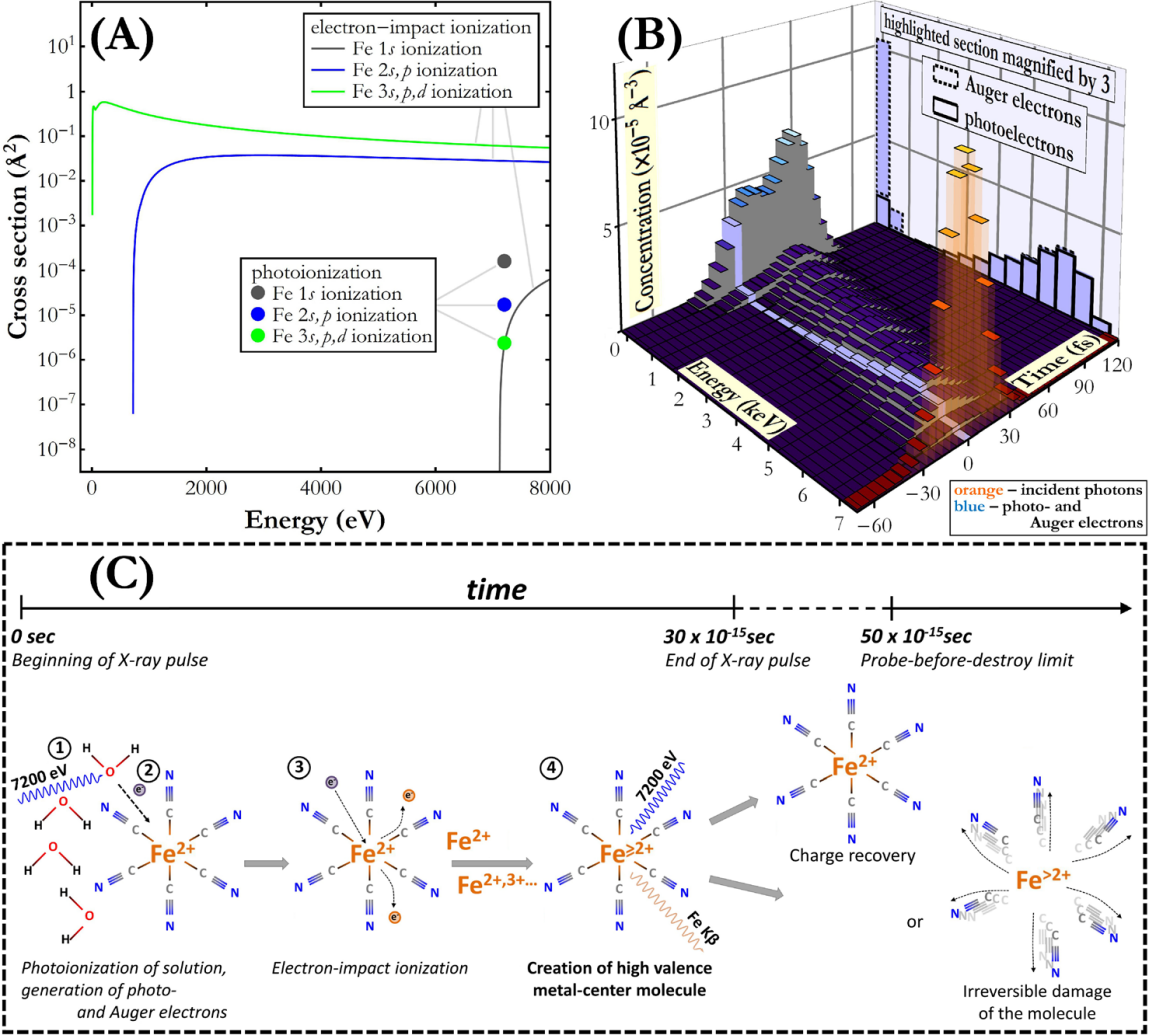
(a) Partial ionization cross sections for the Fe 1*s*, 2*s,p*, and 3*s,p,d* shells through absorption of 7200 eV photons^26^ and through electron-impact.^28^ (b) Simulated energy and time distributions of incident photons as well as energetic photo- and Auger electrons released in the studied material under irradiation with one X-ray pulse with a flux of 244 × 10^30^ photon/(s cm^2^). The data for electrons were integrated over 500 eV energy intervals and 5 fs time periods. The data for photons were integrated over 5 fs time intervals. (c) Illustration of the unique high valence state creation process on the metal site in a Fe(CN)_6_ molecule under ultrashort intense X-ray pulse irradiation.

In order to understand the origin of the observed spectral changes, the fundamental parameters describing photon-matter interactions at the applied experimental conditions were studied. Using the experimental data and the partial photoionization cross sections from Schoonjans *et al.*,^26^ one can calculate that the target sample absorbs 15% of the incident photons and 87% of these photo-absorption events result in O photoionization in the solvent [for more details see supplementary material: General analysis of 7200 eV photons absorption in Fe(CN)_6_/H_2_O solution]. Photoionization and the following Auger decays liberate energetic electrons with different energies. Following the inelastic mean free path data for water reported in Shinotsuka *et al.*,^27^ the travel range of these electrons amounts in average to tens of nm and the electrons may reach the nearest Fe atoms in less than 1 fs. While the total number of generated energetic electrons is on the order of 10^−1^ smaller than that of the incident photons, the cross section for electron-impact Fe ionization is up to the order of 10^4^ larger than the Fe 1*s* shell photoionization cross section, as shown in Fig. 2(a). Moreover, the energetic electrons, unlike photons whose interaction has a binary character, interact with the material multiple times losing the energy gradually. These considerations of fundamental photon- and electron-matter interactions imply that a Fe atom may already have been ionized through electron impact before undergoing the 1*s* shell photoionization and the following Kβ decay.

### Fe atoms' interaction with X-ray photons and X-ray induced energetic electrons

In order to confirm above observations, we performed energy- and time-dependent calculations on electron interactions in the sample within the course of an X-ray pulse (for more details see supplementary material: Simulation of energy and time distribution of energetic electrons). The results of this simulation for the highest photon flux studied in the present work are presented in Fig. 2(b). As shown, photoionization leads to a significant flux of energetic electrons having a very broad energy spectrum, which furthermore changes during the course of X-ray pulse irradiation. The electron spectrum is dominated in the low energy range by Auger electrons and by photoelectrons in the high-energy range. The calculated Fe atom electron-impact ionization frequency (for more details see supplementary material: Simulation of Fe atom's ionization frequency) contains the major contribution of the 3*s,p,d* shell ionization (77%) and of the 2*s,p* shell ionization (23%). The average value of electron-impact ionization frequency reaches more than 43% of the average Fe 1*s* photoionization frequency and in the time range −15 to 15 fs it is 0.003 fs^−1^ while the average photoionization frequency is 0.006 fs^−1^. Hence, there is a considerable probability of electron-impact ionization of the Fe atoms probed by the incident X-ray photons. Before their photoionization and Kβ decay, 5% underwent an extra ionization through interaction with an energetic electron. Precedence of electron-impact ionization is implied by the very short lifetime of the photoinduced Fe 1*s* electron hole [∼0.5 fs (Ref. 26)] making very unlikely the interaction of a Fe atom with an electron between photoionization and the almost immediate Kβ decay. On the other hand, initial Fe 3*s,p,d* and 2*s,p* ionization through electron-impact results in a one electron hole state which quickly evolves through Auger decays forming long-lived many electron hole state.

### Creation of high valence state of the Fe(CN)_6_ molecule

While one the Auger decay ejects one electron from an atom, the entire cascade of such transitions may result in multiple ionization of the atom. To investigate the extent of the influence of Auger decays on the ionization of Fe atoms, all possible Fe fluorescence and Auger decay channels were analyzed (for more details see supplementary material: Simulation of Fe atom's fluorescence and Auger decay cascades). From the multiple electron hole creation probabilities simulated starting with one electron hole at different energy levels, and considering electron-impact ionization frequency for different Fe energy levels, it follows that Fe atoms' ionization due to only the Auger decays induced by a single electron-impact ionization reaches more than 6 electron holes on the valence levels within femtoseconds. The estimated multiple electron hole-states' relative occurrences are approximately 29%: 32%: 21%: 12%: 4%: 2% for 1, 2, 3, 4, 5, and 6 electron holes, respectively.

The analysis of photon and electron interactions unveils the scenario depicted in Fig. 2(c). The incident X-ray pulse interacts with the Fe(CN)_6_/H_2_O solution ionizing mainly O atoms (87% of absorption events), Fe atoms (13% of absorption events), and other atoms (less than 1% of absorption events). The O 1*s* photoionization and the following Auger transitions induce a significant flux of energetic electrons, which are capable of reaching Fe atoms within less than 1 fs. The electron-Fe atom interaction results mainly in Fe 3*s,p,d* shell ionization and the following cascade of Auger decays, lasting a few femtoseconds after the electron-impact ionization event, leads to the ejection of valence electrons from the Fe atom. The Fe(CN)_6_ molecule at this short moment is in a unique state characterized by high valence and preservation of Fe and C atoms' original positions. The intermediate Fe high valence state is then probed by an X-ray photon. In the present study, up to 5% of all the Fe atoms probed were in the high valence state at the moment of interaction with the incident photon. The whole process of the high valence state creation and probe occurs within 30 fs pulse duration, before the molecule movement and irreversible damage [∼50 fs (Ref. 5)] or the much later Fe atom's ground state reestablishment [∼1 ns (Ref. 29)].

### Populations of different Fe oxidation states

To determine the charge state distribution of Fe species, we simulated Fe Kβ X-ray emission spectrum using crystal-field multiplet (CFM) calculations at different oxidation states. The electronic structure of the hexacyanide complexes were evaluated within the density functional theory (DFT) with the B3LYP* functional (15% Hartree-Fock exchange) and a triple-zeta basis set, Lanl2TZ(f) on Fe and 6–311+G(2df) on C and N. Reducing the amount of Hartree-Fock exchange to 10% tested the robustness of the results. The hexacyanoferrate(II) geometry was optimized in a polarized continuum model of water giving Oh symmetry with Fe-C bonds' distances of 1.939, which is within 0.02 Å of the distances in the crystal structure. The electron-loss products were calculated with all possible spin multiplicities and then allowing for electronic relaxation to the most stable electron configuration, which in all cases was the state with the highest multiplicity. No geometry relaxation or solvent equilibration was allowed. Electron densities were allowed to break symmetry and were checked for internal instabilities. DFT calculations were performed using Gaussian09.D01.^30^ Kβ emission spectra of Fe^2+^ to Fe^5+^ were calculated within the crystal-field multiplet model using the CTM4XAS program.^31^ The ligand-field parameter 10Dq was set to 4.2 eV for hexacyanoferrate(II).^32^ Simulations of Kβ spectra for other oxidation states were done both with constant 10Dq for all oxidation states, as well as with individually varying 10Dq as detailed in the supplementary material: Details of DFT calculations. Spectra were broadened with a Lorentzian with a full width at half maximum (FWHM) of 1.5 eV. The contribution from the spectrometer resolution was on the order of 0.4 eV; thus, it is negligible comparing to the natural core-hole lifetime.

**FIG. 3.**
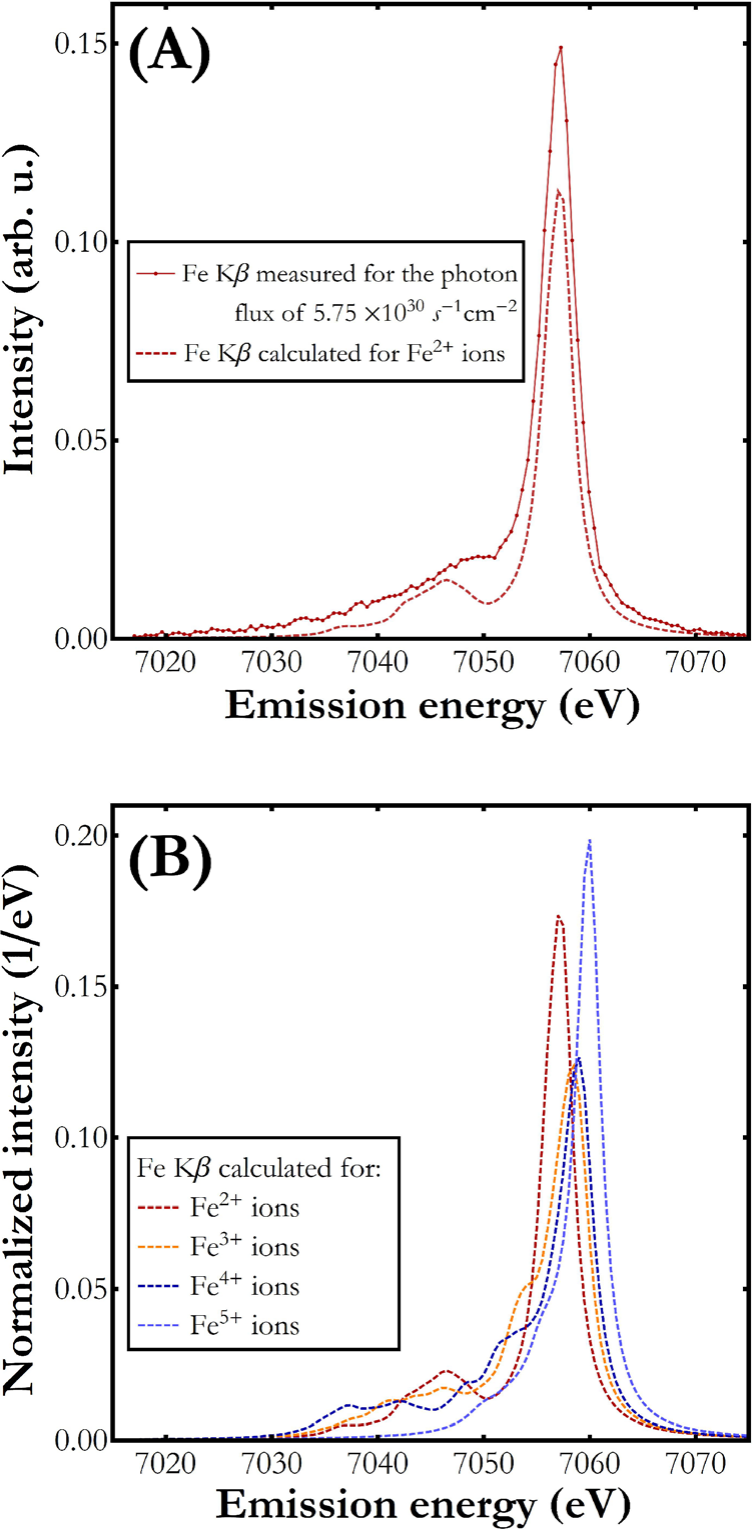
(a) The Fe Kβ emission signal calculated (dashed line) and measured (dots connected with solid line) for the Fe(CN)_6_/H_2_O sample. (b) The Fe Kβ emission spectrum calculated for Fe(CN)_6_/H_2_O for different Fe oxidation states from 2+ up to 5+. The spectra were normalized to unit area for integrated area difference (IAD) analysis.^23^

Figure 3(a) presents a comparison of the Fe Kβ X-ray emission spectrum measured for the lowest incidence photon flux with that of hexacyanoferrate(II). The calculated spectrum describes well the shape of the measured Fe Kβ emission spectrum including the main Kβ_1,3_ transition as well as the low energy satellite line. In order to confirm the experimental spectral difference at higher incidence X-ray fluxes and the expected effect of high valence Fe state creation, the calculations were performed for a Fe(CN)_6_ molecule also at higher Fe oxidation states. The result of the computation is presented in Fig. 3(b). As shown, higher oxidation state at the Fe site leads to a shift of the spectral curve toward higher energy. Simultaneously, a strong variation of the spectral shape can be seen with increasing Fe oxidation state. The observed variation between the main Kβ_1,3_ emission line and the Kβ′ feature is caused by changes of the relative population of the high spin and the low spin electron configurations among Fe atoms. The lowest population of the high spin final state is observed for 3+ and 4+ oxidation states.

**FIG. 4.**
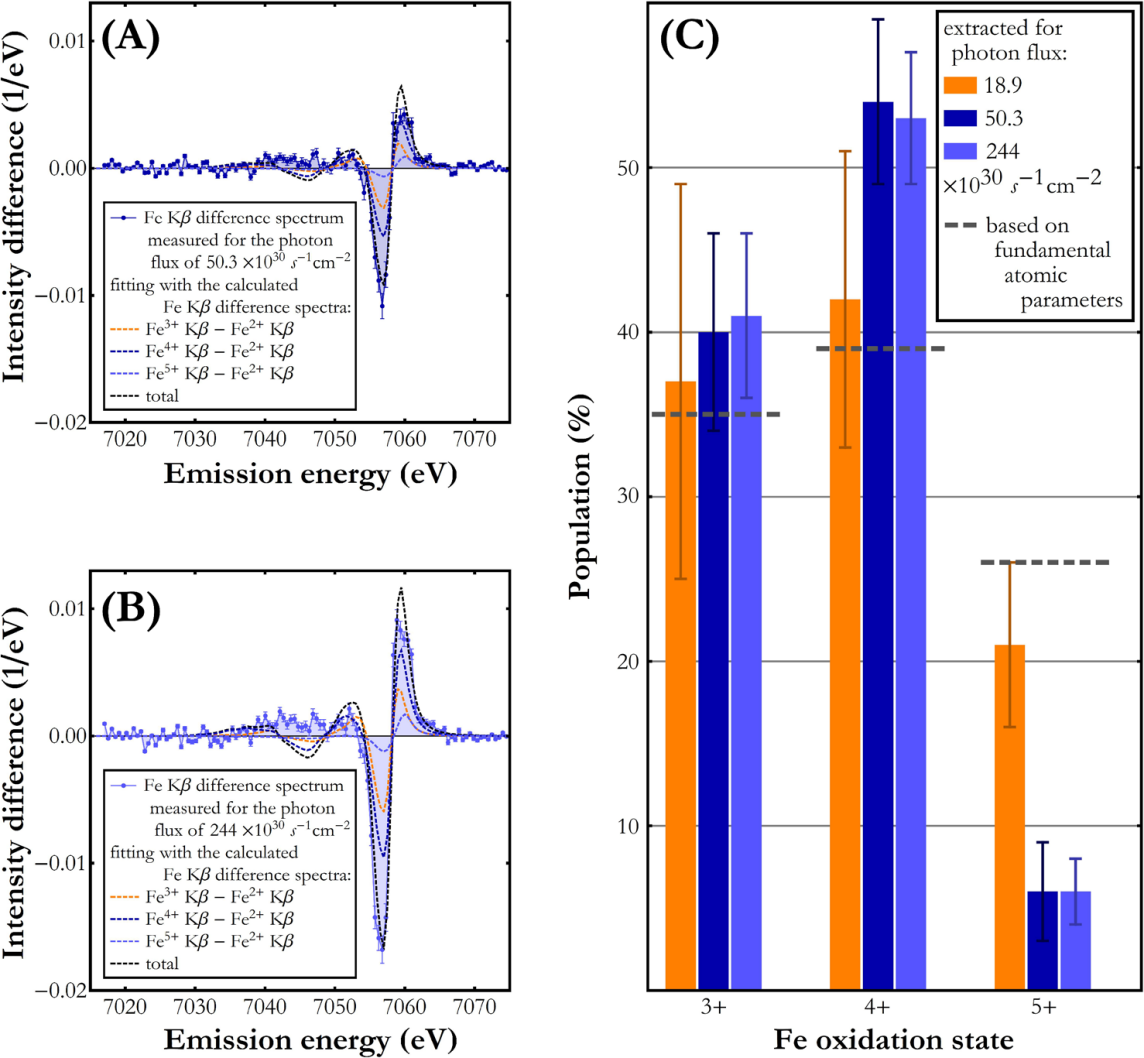
Fitting a linear combination of the calculated difference spectra to the difference spectra measured for the photon flux of (a) 50.3 × 10^30^ photon/(s cm^2^) and for the photon flux of (b) 244 × 10^30^ photon/(s cm^2^). Only the experimental data points in the energy range 7048 eV–7068 eV were considered in the fitting procedure. The fit components corresponding to the Fe^3+^-Fe^2+^, Fe^4+^-Fe^2+^, and Fe^5+^-Fe^2+^ spectral changes are plotted as orange, dark blue, and light blue dashed lines, respectively. A black dashed line marks the total of the fit components' linear combination. (c) Relative populations of Fe oxidation states 3+, 4+, and 5+.

The calculated Fe^3+^, Fe^4+^, and Fe^5+^ Kβ X-ray emission difference spectra, obtained through subtraction of the calculated spectra shown in Fig. 3(b) from the Fe^2+^ one, were used to estimate the relative Fe^3+^, Fe^4+^, and Fe^5+^ oxidation states' populations through fitting their linear combination to the experimental difference spectra. The best-fit results for the two highest photon fluxes studied are shown in Figs. 4(a) and 4(b). As demonstrated, the observed spectral differences can be well reproduced using the three calculated Fe^3+^-Fe^2+^, Fe^4+^-Fe^2+^, and Fe^5+^-Fe^2+^ difference spectra. The theoretical curves describe well both the negative and the positive signal differences around 7058 eV and 7060 eV, respectively. The relative occurrences of Fe oxidation states extracted from the fit parameters are presented in Fig. 4(c). The difference signal increases for higher X-ray flux but the spectral composition remains the same with about 30% contribution from each of the Fe^3+^, Fe^4+^, and Fe^5+^ oxidation states. The Fe oxidation states' populations obtained from the difference spectra fit are very similar to the estimates based on the fundamental atomic parameters [see Creation of high valence state of the Fe(CN)_6_ molecule] where the expected relative populations, assuming only the three Fe^3+^, Fe^4+^, and Fe^5+^ charge states contributions, were 35%, 39%, and 26%, respectively. We wish to emphasize the lack of reference experimental Fe^4+^ and Fe^5+^ Kβ spectra which precludes the calculated spectra verification. This circumstance and the insufficient quality of the spectra measured in this work do not allow credible determination of the eventual dependence of Fe oxidation states' populations on incident X-ray flux. Nevertheless, the presented results prove the formation of higher Fe oxidation states under X-ray pulse irradiation and indicate their distribution maximum at Fe^4+^.

### Mechanism of the high valence state creation

**FIG. 5.**
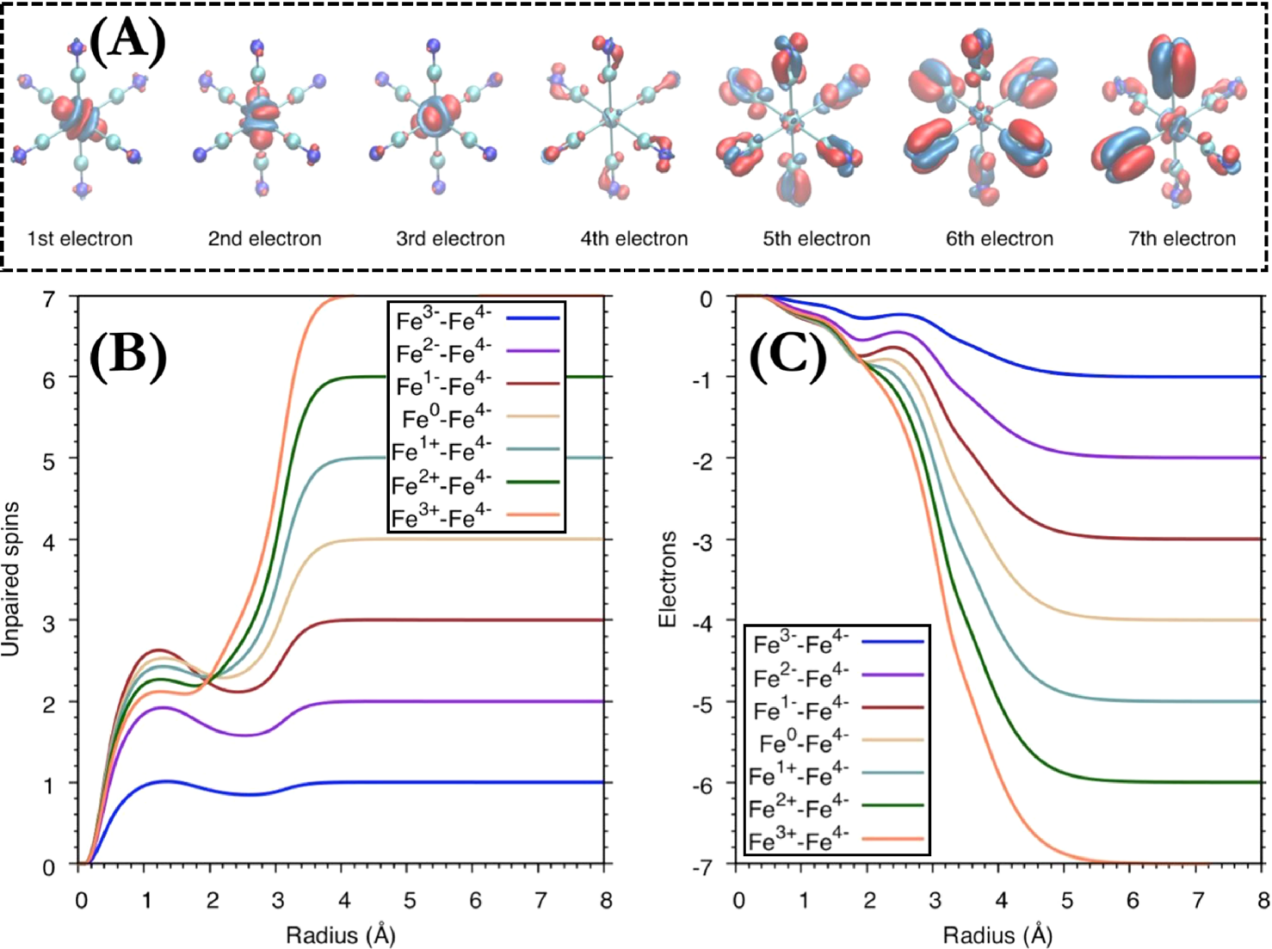
(a) Charge density difference maps upon subsequent electron loss from hexacyanoferrate(II) molecule. Negative values (electron loss) are shown in red and positive values (electron gain) in blue. The surface isovalues are ±0.008. (b) Integrated radial spin density (RSD) and (c) integrated radial charge density (RCD) differences for electron loss from hexacyanoferrate(II).

The DFT calculations allow us furthermore to propose a mechanism for the creation of high valence states from the perspective of the molecular electronic structure. The changes in electron density during the electron-loss steps are shown in Fig. 5(a). Starting from a hexacyanoferrate(II) molecule, the first electron is removed from a metal-dominated t_2g_ orbital (red lobe), which results in the formation of ferricyanide. At the same time, the electron density increases along the Fe-C bonds, a consequence of the increased sigma donation from the CN ligand. Differences in radial spin density (RSD) and radial charge density (RCD) are shown in Figs. 5(b) and 5(c). From the RSD, it is clear that one electron is removed from iron, while the changes in the RCD are more gradual due to ligand polarization that compensates the loss of the t_2g_ electron. The next two steps are similar to the first one, with metal t_2g_-centered oxidations; see Fig. 5(a). Removing three electrons, thus, leads to an Fe(V) oxidation state. After the third electron has been removed, the oxidations are ligand-centered. This can be seen from the marginal change in both RSD and RCD at the metal. When additional electrons are removed, there is a gradual loss of spin on iron, but this is accompanied by an electron flow from the ligands to the metal. The system, thus, approaches an Fe(IV) species rather than an Fe(VI) when more electrons are lost. The flow of charge density from ligand to metal upon creation of a ligand hole is consistent with observations for the ligand-to-metal charge-transfer state of ferricyanide.^33^ The conclusion that the Fe(V) state is the highest accessible oxidation state does not change when all possible spin multiplicities are considered, or when changing the density functional (for more details see supplementary material: Details of DFT calculations).

## CONCLUSIONS

In summary, X-ray photons induce solvent ionization within sub-fs leading to the formation of solvated electrons. Following the inelastic mean free path data for water, the travel range of these electrons amounts in average to tens of nm and the electrons may reach the nearest Fe atoms in less than 1 fs and catalyze coulombic interactions without affecting molecule's atomic positions. The creation of short-lived high valence states is an unexpected phenomenon on the basis of the probe-before-destruction methodology, a strategy pioneered for structure-related measurements free of beam damage. However, the methodology was devised to outrun atoms movement (>50 fs), which is the prime event affecting the resolution in structure-related measurements.

This work has direct and immediate implications on X-ray spectroscopy studies. First, it shows that the probed atom's electronic structure investigated in measurements on embedded samples using even ultrashort XFEL pulses may be affected by the energetic electrons released through the photoionization of the atom's environment. As it is known, the X-ray damage mechanisms are indeed observed in many laboratory and synchrotron experiments within time scale ranging from minutes to days, depending on sample composition and applied X-ray intensity. In this work, under typical operational conditions of an XFEL machine, we induced and detected X-ray damage within the course of a single X-ray pulse. The damage will be inherently induced whenever the sample is exposed to X-rays with the number of damaged molecules proportional to the applied X-ray flux and, thus, to the absorbed X-ray dose. It will also occur under photon fluxes lower than the ones investigated in this work although, due to the low amount of damaged species, it can be beyond detection limit and may require longer irradiation times to be detected. Fortunately, the extent of the damaged species can be assessed and corrected for by analysis of the measured XES spectra. Second, it delivers a methodology for the creation and on-the-fly probing of synthetically inaccessible states of matter, which are of interest in biological and chemical catalysis (transition state species)^34^ and in astrochemistry. Note that the proposed methodology affords a certain degree of selectivity and control of the process because the damage process can be predicted, on the basis of solvent used, and the electronic changes are induced much faster than direct photoabsorption process occurs.

## SUPPLEMENTARY MATERIAL

See supplementary material for more details on the analysis of X-ray pulse interaction with the sample, kinetic model used in simulation of the free electron production and interaction with matter, Monte Carlo simulation of Fe atoms' Auger decays, and the DFT calculations used in determining the Fe high valence state creation mechanism.
